# The anti-vascular endothelial growth factor receptor-1 monoclonal antibody D16F7 inhibits invasiveness of human glioblastoma and glioblastoma stem cells

**DOI:** 10.1186/s13046-017-0577-2

**Published:** 2017-08-10

**Authors:** Maria Grazia Atzori, Lucio Tentori, Federica Ruffini, Claudia Ceci, Lucia Lisi, Elena Bonanno, Manuel Scimeca, Eskil Eskilsson, Thomas Daubon, Hrvoje Miletic, Lucia Ricci Vitiani, Roberto Pallini, Pierluigi Navarra, Rolf Bjerkvig, Stefania D’Atri, Pedro Miguel Lacal, Grazia Graziani

**Affiliations:** 1 Department of Systems Medicine, University of Rome Tor Vergata, Via Montpellier 1, 00133 Rome, Italy; 2 Laboratory of Molecular Oncology, “Istituto Dermopatico dell’Immacolata”-IRCCS, Via dei Monti di Creta, 104, 00167 Rome, Italy; 3 Istituto di Farmacologia, Università Cattolica del Sacro Cuore, Largo Francesco Vito 1, 00168 Roma, Italia; 4 Department of Experimental Medicine and Surgery, University of Rome Tor Vergata, Rome, Italy; 5 Department of Genomic Medicine, University of Texas MD Anderson Cancer Center, Houston, TX USA; 6 INSERM U1029, University of Bordeaux, Pessac, France; 7 Department of Biomedicine, University of Bergen, Bergen, Norway; 8 Department of Hematology, Oncology and Molecular Medicine, “Istituto Superiore di Sanità” (ISS), Rome, Italy; 9 Department of Neurosurgery, Università Cattolica del Sacro Cuore, Rome, Italy; 10 UOC di Farmacologia, Fondazione Policlinico Universitario Agostino Gemelli, Largo Francesco Vito 1, 00168 Roma, Italia

**Keywords:** VEGFR-1, PlGF, VEGF-A, Glioblastoma, Angiogenesis, Molecular marker, Molecular medicine

## Abstract

**Background:**

Glioblastoma (GBM) is a highly migratory, invasive, and angiogenic brain tumor. Like vascular endothelial growth factor-A (VEGF-A), placental growth factor (PlGF) promotes GBM angiogenesis. VEGF-A is a ligand for both VEGF receptor-1 (VEGFR-1) and VEGFR-2, while PlGF interacts exclusively with VEGFR-1. We recently generated the novel anti-VEGFR-1 monoclonal antibody (mAb) D16F7 that diminishes VEGFR-1 homodimerization/activation without affecting VEGF-A and PlGF binding.

**Methods:**

In the present study, we evaluated the expression of VEGFR-1 in human GBM tissue samples (*n* = 42) by immunohistochemistry, in cell lines (*n* = 6) and GBM stem cells (GSCs) (*n* = 18) by qRT-PCR and/or western blot analysis. In VEGFR-1 positive GBM or GSCs we also analyzed the ability of D16F7 to inhibit GBM invasiveness in response to VEGF-A and PlGF.

**Results:**

Most of GBM specimens stained positively for VEGFR-1 and all but one GBM cell lines expressed VEGFR-1. On the other hand, in GSCs the expression of the receptor was heterogeneous. D16F7 reduced migration and invasion of VEGFR-1 positive GBM cell lines and patient-derived GSCs in response to VEGF-A and PlGF. Interestingly, this effect was also observed in VEGFR-1 positive GSCs transfected to over-express wild-type EGFR (EGFRwt^+^) or mutant EGFR (ligand binding domain-deficient EGFRvIII^+^). Furthermore, D16F7 suppressed intracellular signal transduction in VEGFR-1 over-expressing GBM cells by reducing receptor auto-phosphorylation at tyrosine 1213 and downstream Erk1/2 activation induced by receptor ligands.

**Conclusion:**

The results from this study suggest that VEGFR-1 is a relevant target for GBM therapy and that D16F7-derived humanized mAbs warrant further investigation.

**Electronic supplementary material:**

The online version of this article (doi:10.1186/s13046-017-0577-2) contains supplementary material, which is available to authorized users.

## Background

Vascular endothelial growth factor receptor-1 (VEGFR-1) is a high-affinity tyrosine kinase receptor for VEGF-A, VEGF-B, and placental growth factor (PlGF) ligands [1, 2]. VEGFR-1 is composed of seven extracellular immunoglobulin homology domains, a single transmembrane region, and an intracellular tyrosine kinase domain. The interaction of VEGFR-1 with its ligands induces receptor dimerization, tyrosine auto-phosphorylation, transphosphorylation, and docking of signaling proteins [1–3]. VEGFR-1 also exists as soluble form that acts as decoy receptor preventing VEGF-A and PlGF interaction with transmembrane receptors [3]. While VEGFR-1 does not play a relevant role in physiological angiogenesis in the adult, this receptor is indeed important in tumor angiogenesis and directly activates signaling pathways crucial for tumor growth, progression, and metastasis in cancer cells [4, 5].

VEGF-A binds to both VEGFR-1 and VEGFR-2, while VEGF-B and PlGF interact exclusively with VEGFR-1. VEGF-A is the most widely studied angiogenic factor, and its role in tumor angiogenesis via stimulation of VEGFRs expressed on tumor endothelium is well established [6]. It also directly interacts with VEGFRs expressed on cancer cells stimulating disease progression. In its homo- or heterodimeric form with PlGF, VEGF-A may activate VEGFR-1 and VEGFR-2 homo- or heterodimers [7, 8]. PlGF binds to VEGFR-1 with higher or lower affinity compared with VEGF-B or VEGF-A, respectively [4, 9]. VEGF-B role in tumor biology appears limited [10], while PlGF seems to have an important disease-associated role because its expression, which is low or undetectable in most adult healthy tissues, is significantly up-regulated in a number of pathological conditions including cancer [5]. Interestingly, PlGF is produced by tumor, endothelial, and other cells of the tumor stroma including inflammatory cells promoting migration, proliferation, and survival [11, 12]. Moreover, high tumor expression levels are associated with a poor prognosis [11, 12].

VEGFR-1 is expressed in endothelial cells during vessel formation and remodeling, macrophages, myoepithelial cells, and a variety of human cancer cells, favoring cell migration and survival [1, 2]. In tumors, VEGFR-1 signaling inhibits apoptosis, induces chemoresistance, and predicts poor prognosis and recurrence [1, 13, 14]. Moreover, it is involved in the mobilization of myeloid bone marrow-derived cells that generate tumor-associated macrophages [1, 15]. VEGF-A and PlGF binding to VEGFR-1 can induce phosphorylation and activation of mitogen-activated protein kinases (MAPKs) Erk1/2 and p38 [16], and through VEGFR-1 activation, PlGF also stimulates the trans-phosphorylation of specific VEGFR-2 tyrosine residues [17]. Interestingly, it has been proposed that PlGF may enhance tumor cell invasiveness by augmenting matrix metalloproteinase (MMP) secretion via Erk1/2 signaling [18].

A number of studies have been designed to disrupt tumor angiogenesis and growth by anti-VEGF-A and anti-VEGFR-2 monoclonal antibodies (mAbs) or VEGFRs small molecule tyrosine kinase inhibitors. We hypothesize that molecules selectively targeting VEGFR-1 may inhibit tumor vascularization and invasion/metastasis while producing lower systemic toxicity than agents directed against VEGF-A or VEGFR-2, which cause adverse effects due to inhibition of physiological angiogenesis [19]. Therefore, we have generated an anti-VEGFR-1 mAb (D16F7) by immunizing mice with a peptide corresponding to amino acids 149–161 of human VEGFR-1 [15]. While not affecting binding of VEGF-A and PlGF, D16F7 reduces VEGFR-1 homodimerization and activation by both ligands. This mAb inhibits chemotaxis of human endothelial, myelomonocytic, and melanoma cells in response to VEGFR-1 ligands. In an in vivo murine model, D16F7 is well tolerated, inhibits angiogenesis in response to inflammatory stimuli and markedly affects melanoma growth. The antitumor effect is associated with tumor cell apoptosis, vascular abnormalities, reduced monocyte/macrophage infiltration and impaired myeloid progenitor mobilization [15].

In the present study we investigated whether D16F7 exerts inhibitory activity against human glioblastoma (GBM), which is a highly aggressive brain tumor that relies on angiogenesis for growth and histological progression [20, 21]. The standard care of newly diagnosed GBM includes surgical tumor resection followed by radiation therapy and chemotherapy with the alkylating agent temozolomide [22, 23]. GBM exhibits a highly abnormal blood supply, which leads to swelling and reduced blood perfusion within the tumor and causes it to become resistant to chemo- and radiotherapy. VEGF-A and PlGF expression by glioma cells additionally induces accumulation of VEGFR-1–positive bone marrow-derived myeloid cells in the tumor tissue [24]. While anti-VEGF-A treatment has become part of standard post-surgical treatment for recurrent GBM, its beneficial effects are temporary and it does not effectively extend patient overall survival [25].

In this context, our results demonstrate that D16F7 markedly inhibits chemotaxis and invasiveness of GBM cells and patient-derived GBM stem cells (GSCs) in response to VEGF-A and PlGF, suggesting that VEGFR-1 might represent a suitable target that deserves further investigation for GBM treatment.

## Methods

### Immunohistochemical analysis of VEGFR-1 in tissue samples from GBM patients

We enrolled 42 adults [mean age 60.51 (34–79), 27 males/15 females], who underwent surgery for primary GBM at the Institute of Neurosurgery, “Università Cattolica del Sacro Cuore” (Rome, Italy), from March 2005 to September 2011. Diagnosis of GBM was established on histological examination according to the WHO classification (grade IV) of tumors of the nervous system. All patients provided written consent to use their specimens for research and the research proposal was approved by the university Ethical Committee. Tissues were fixed in 4% paraformaldehyde in 0.1 M phosphate buffer pH 7.6 at 4 °C overnight. Tissues were rehydrated in graded ethanol solutions, xylene and finally embedded in Paraplast Plus (Tyco/Healthcare, Mansfield, MA). Sections, 3–4 μm thick, were deparaffinized and incubated in 10 mM citrate buffer, pH 6.0, dry heated for 10 min each to unmask antigen sites, cooled and washed in phosphate-buffered saline (PBS). Endogenous peroxidase activity was inhibited by rinsing the slides in 3% hydrogen peroxide for 5 min. Nonspecific binding was blocked by 5 min incubation with the Super Block Solution (ScyTek Laboratories, UT). After washing in PBS, sections were incubated for 10 min at room temperature with rabbit anti-Human Flt-1/VEGFR-1 polyclonal antibody (1:50; Spring Bioscience, Pleasanton, CA). The immunostaining conditions were standardized using human placenta as positive control (data not shown). Sections were washed extensively with PBS and subsequently processed using the Ultra Tek Anti-Polyvalent kit (ScyTek Laboratories). Finally sections were treated with 3,3′-diaminobenzidine as chromogen, contrasted with hematoxylin and mounted [26]. Two blinded examiners evaluated staining of human tumor specimens. For each specimen, the number of VEGFR-1 positive cells in total 50 cells was counted.

### Cell lines and culture conditions

The human GBM cell lines A172, U87, LN18, T98G and U373 were from American Type Culture Collection (ATCC, Manassas, VA). Cells were maintained in DMEM (Sigma-Aldrich, St. Louis, MO) supplemented with 10% fetal bovine serum (FBS, Sigma-Aldrich), 2 mM L-glutamine, 100 units/ml penicillin, and 100 μg/ml streptomycin sulfate, at 37 °C in a 5% CO_2_ humidified atmosphere.

GSCs were isolated from 18 surgical samples of adult patients who had undergone craniotomy at the Institute of Neurosurgery, “Università Cattolica del Sacro Cuore” (Rome, Italy). Prior to surgery all patients provided written informed consent according to the Declaration of Helsinki and the research proposal was approved by the university Ethical Committee. In regard to GSCs origin, the diagnosis of GBM was established on histological examination according to the WHO classification (grade IV) of tumors of the nervous system. Tumor samples were subjected to mechanical dissociation. The resulting cell suspension was cultured in a serum-free medium supplemented with 20 ng/ml epidermal growth factor (EGF) and 10 ng/ml FGF-2 (PeproTech, Rocky Hill, NJ). Generation of GSCs was defined by the following criteria: in vitro formation of primary neurospheres expressing stem cell markers such as CD133, SOX2, Musashi-1 and nestin, capacity of self-renew, ability to co-express astrocytic as well as neuronal phenotypic markers after serum-induced differentiation in vitro [27–29]. GSCs were characterized by immunofluorescence analysis as previously described [30]. All the GSC lines tested in this study were positive for SOX2, Musashi-1 and nestin, whereas they expressed different levels of CD133 (data not shown).

P3, EGFRwt^+^, and EGFRvIII^+^ GSC lines were previously described [31]. Cells were cultured in neurobasal medium (NBM) supplemented with 2 mM GlutaMAX and 1× B-27 (Life Technologies, Carlsbad, CA), 1× penicillin/streptomycin (Lonza, Basel, Switzerland), 1 U/ml heparin (Sigma-Aldrich) and 20 ng/ml FGF-2 (hereafter referred to as complete NBM).

The human umbilical vascular endothelial cells (HUVEC) were isolated from freshly delivered umbilical cords as previously described [32] and cultured in EGM-2.

The human GR-Mel and M14 melanoma cell lines, used as positive and negative controls for VEGFR-1 or VEGFR-2 transcripts, were obtained and cultured as previously described [33].

Human GBM cell lines were authenticated by STR profiling (BMR genomics, Padova, Italy) and GSCs lines were periodically tested for the expression of phenotypic markers [in P3-derived cells, EGFR amplification or mutation; in GSCs, the above described markers].

### Generation of GBM cell lines overexpressing VEGFR-1

Cell clones were obtained by limiting dilution from U87 cells and one clone was transfected with the pBLAS49.2 or pBLAS49.2/VEGFR-1 plasmids. The pBLAS49.2/VEGFR-1 construct was obtained by cloning of VEGFR-1 cDNA from pcDNA3/VEGFR-1 plasmid (a generous gift of Dr. K. Ballmer-Hofer, PSI, Zurich) into pBLAS49.2 vector (InvivoGen, San Diego, CA). Transfection was performed using lipofectamine 2000 (Invitrogen, Camarillo, CA), as described by the manufacturer, and transfected cells were selected in blasticidine (Invitrogen) containing culture medium. Antibiotic resistant clones were isolated by ring cloning and U87 clones maintained in the presence of 2.5 μg/ml blasticidine. VEGFR-1 expressing subclones were identified by RT-PCR and Western blotting.

### Analysis of VEGFRs transcripts

Quantification of membrane VEGFR-1 and VEGFR-2 transcripts was performed by quantitative real-time reverse transcriptase –polymerase chain reaction (qRT-PCR) according to the dual-labeled fluorigenic probe method and using an ABI Prism 7000 sequence detector (PerkinElmer, Groningen, the Netherlands), as previously described [34]. Expression levels were calculated by the relative standard curve method. Primers used were as follows: VEGFR-1, forward 5′-ACCGAATGCCACCTCCATG-3′ and reverse 5′-AGGCCTTGGGTTTGCTGTC-3′; VEGFR-2, forward 5′-GTCTATGCCATTCCTCCCCC-3′ and reverse 5′-GAGACAGCTTGGCTGGGCT-3′. For each sample, the level of VEGFR-1 or VEGFR-2 transcripts was normalized to that of 18S RNA (TaqMan® Gene Expression Assay, Applied Biosystems, Foster City, CA) and referred to the values of the VEGFR-1 and VEGFR-2 negative M14 cell line, to which the arbitrary value of 1 was assigned.

In VEGFR-1-transfected cells detection of VEGFR-1 transcript was confirmed by RT-PCR analysis. The cDNA preparation followed by PCR amplification to evaluate VEGFR-1 expression was performed as previously described [35], utilizing an annealing temperature of 58 °C and the following primers: human VEGFR-1, forward primer 5′-CTCCTGAGTACTCTACTCCT-3′, reverse primer 5′-GAGTACAGGACCACCGAGTT-3′ (640 bp fragment); human glyceraldehyde-3-phosphate dehydrogenase (GAPDH), forward primer 5′-TCCCATCACCATCTTCCA-3′, reverse primer 5′-CATCACGCCACAGTTTCC-3′ (380 bp fragment).

### Quantification of VEGF-A and PlGF in GBM cell culture conditioned media by ELISA

Conditioned media from GBM cells were obtained by incubating semi-confluent cultures for 24 h in 0.1% BSA/DMEM medium without FBS. These conditions did not significantly affect cell viability. Supernatants were concentrated at least 10-fold in Centriplus concentrators (Amicon, Beverly, MA). Cells were detached from the flasks with PBS/EDTA. Cytokine secretion values were normalized by the total number of cells.

Quantification of the amount of VEGF-A and PlGF in the conditioned medium was performed using goat anti-VEGF-A or anti-PlGF IgGs (R&D Systems, Abingdon, UK), at a concentration of 10 μg/ml in PBS, to coat Maxisorp Nunc immunoplates (Nunc, Roskilde, Denmark). Detection of the cytokines was performed with biotinylated goat anti-VEGF or anti-PlGF IgGs (0.4 μg/ml; R&D Systems) followed by incubation with streptavidin alkaline phosphatase conjugate (1:10,000) (Roche, Monza, Italy) and alkaline phosphatase reaction. Optical density at 405 nm was measured in a 3550-UV Microplate reader (Bio-Rad, Hercules, CA).

### Western blotting

Proteins were run in 10% SDS-polyacrylamide gels and transferred to supported nitrocellulose membranes by standard techniques. Immunodetection was performed using the following antibodies: mouse monoclonal anti-VEGFR-1 (clone D2, 1:500; Santa Cruz Biotechnology, Santa Cruz, CA); mouse monoclonal anti-EGF receptor (EGFR) (528, 1:1000; Santa Cruz Biotechnology); mouse monoclonal antibody anti-EGFRvIII (L8A4; 1:1000; Absolute Antibody, Oxford, UK); rabbit polyclonal anti-phosphorylated VEGFR-1 at tyrosine 1213 (1:500; R&D Systems); rabbit polyclonal anti-Erk1&2 (1:1000; Genetex, Irvine, CA); rabbit polyclonal anti-phospho-Erk1&2 (Thr/Tyr185/187, 1:1000; Invitrogen); or rabbit polyclonal anti-β-actin (1:10,000; Sigma Aldrich) primary antibodies. Anti-mouse or anti-rabbit Ig/Horseradish peroxidase secondary antibodies and ECL Western blotting detection reagents from GE Healthcare (Milan, Italy) were used to identify the proteins of interest.

### Chemotaxis assay and spheroid invasion assay

In vitro migration assay was performed using Boyden chambers equipped with 8 μm pore diameter polycarbonate filters (Nuclepore, Whatman Incorporated, Clifton, NJ) coated with 5 μg/ml gelatin (Sigma-Aldrich), as previously described [36, 37]. Treatment with D16F7 was carried out by incubating the cells in the presence of the indicated mAb concentrations in a rotating wheel for 30 min at room temperature. Cells (2 × 10^5^/chamber) were then loaded in the upper compartment of Boyden chambers and migration assay, toward stimuli (50 ng/ml VEGF-A or PlGF) present in the lower compartment, was done in the absence or in the presence of D16F7 mAb or, in selected experiments, of an equivalent amount of a species- and isotype-matched control antibody (mouse IgG1, R&D Systems) for 18 h. Migrated cells, attached to the lower side of the filters, were stained with crystal violet counted in triplicate samples for a total of 12 high power (200× magnification) microscopic fields.

For spheroid invasion assay, tumor cells (25,000–30,000 cells/ml) were suspended in DMEM-1640 containing 10% FBS (for GBM cell lines) or in complete NBM (for P3-derived GSC lines), supplemented with methyl cellulose (0.24% final concentration; Sigma-Aldrich), seeded in 96-well round bottom cell culture plates (100 μl/well; Corning® Costar® Ultra-Low attachment multi-well, Sigma-Aldrich) and centrifuged at 3000 rpm for 90 min [31]. Plates were then incubated for 24 h under standard culture conditions (5% CO_2_, at 37 °C) to allow spheroid formation. Spheroids were collected, embedded individually in 100 μl of matrigel (reduced growth factor basement membrane matrix, Pathclear, Cultrex, Gaithersburg, MD) in 0.1% BSA/DMEM or NBM medium, with or without VEGF-A or PlGF (50 ng/ml) and/or D16F7 mAb, and plated in each well of a 96-well flat bottom plate, previously coated with 50 μl of matrigel. Five to ten replicates were set up for each experimental group. After matrigel solidification at 37 °C, 100 μl of invasion medium, with or without VEGF-A, PlGF or EGF (50 ng/ml), were added and plates incubated at 37 °C for up to 72 h. Spheroids were visualized and photographed using a Nikon Eclipse TS100 microscope in conjunction with a Nikon DS-Fi1 high resolution camera (Melville, NY). Measurements were performed using Adobe Photoshop CS6 software. Relative invasion area was defined as area of spheroids (in mm^2^) at each time point minus area on day 0.

Preliminary experiments on U87 and U87-MF24 cell migration in response to PlGF and in the presence of graded concentrations of D16F7 indicated that the IC_50_ values were 1.54 ± 0.22 μg/ml and 2.49 ± 0.56 μg/ml, respectively. Therefore, based on these results we selected the mAb concentrations to be tested in the functional assays.

### Cell proliferation assay

Cell proliferation was evaluated in 96-well plates using the tetrazolium compound MTS [3-(4,5-dimethylthiazol-2-yl)-5-(3-carboxymethoxyphenyl) 2-(4-sulphophenyl)-2H–tetrazolium, inner salt] from Promega (Madison, WI), as previously described [38]. Briefly, increasing numbers of GBM cells, suspended in complete medium containing graded concentrations of D16F7 up to 20 μg/ml of D16F7 or control antibody or without antibodies, were dispensed into flat-bottom 96-well plates and grown at 37 °C in a 5% CO_2_ humidified atmosphere. Six replica wells were used for every condition. After 3 days, 20 μl of MTS solution were added to each well and cells were incubated at 37 °C for 2 h. Absorbance was read at 490 nm (reference wavelength 655 nm) using a 3550-UV Microplate reader (Bio-Rad).

### Statistical analyses

Statistical analysis of the differences between pairs of groups was performed by Student’s *t* test. For multiple comparisons ANOVA analysis, followed by Bonferroni’s post-test, was used. Statistical significance was determined at α = 0.05 level. Differences were considered statistically significant when *p* < 0.05.

## Results

### Analysis of VEGFR-1 in patient-derived GBM specimens and cell lines

To investigate the relevance of VEGFR-1 in GBM, we initially investigated the expression of VEGFR-1 by immunohistochemistry in tissue specimens obtained from 42 adult GBM patients (Table 1). Most of GBM tissue samples showed a significant VEGFR-1 immunoreactivity. VEGFR-1 staining was observed in association with GBM cells as well as with endothelial cells (Table 1 and Additional file 1: Figure S1 and Additional file 2: Figure S2) in accordance with previous studies [39, 40].

**Table 1:** Characteristics and VEGFR-1 expression of GBMs from which tissue specimens were derived

GBM patient	Tumor location	Primary (P) Recurrent (R)	Overall Survival (months)	VEGFR-1^a^
1	NA	P	13	+++
2	Temporal	P	19	+
3	Temporal	P	60	−
4	Frontal	R	NA^b^	++
5	Frontal	P	7	+++
6	NA	P	NA	+++
7	Frontal	P	15	+++
8	Frontal	P	2	+++
9	Temporal	P	4	+++
10	Occipital	P	33	+
11	Temporal	P	14	−
12	NA	P	NA	+
13	NA	R	NA	+
14	Temporal	P	53	+
15	Parietal	P	53	+
16	NA	P	9	+++
17	Frontal	P	NA	+
18	Frontal	P	NA	+++
19	Tempo-Parietal	R	53	+++
20	Temporal	P	NA	+++
21	NA	P	NA	+++
22	Temporal	P	NA	+
23	Parietal	P	6	+++
24	NA	P	NA	++
25	Temporal	P	NA	+++
26	NA	R	NA	+
27	Frontal	R	NA	+
28	Occipital	P	NA	+++
29	NA	P	NA	+++
30	Temporal	P	NA	+++
31	NA	P	NA	++
32	Temporal	P	NA	+++
33	Temporal	P	6	+
34	NA	P	NA	+++
35	Frontal	P	12	+
36	Fronto-Temporal	P	NA	++
37	Occipital	P	8	+++
38	Frontal	P	NA	−/+
39	Temporal	P	NA	+++
40	NA	P	38	−/+
41	NA	R	24	++
42	Frontal	P	12	+

^a^The number of positive VEGFR-1 cells in the tumor mass was counted in a total of 50 cells.VEGFR-1 staining was scored as percentage of positively stained cells: -, <10%; −/+, 11–25%; +, 26–50%; ++, 51–75%; +++, >75%

^b^NA: not available

**Fig. 1:**
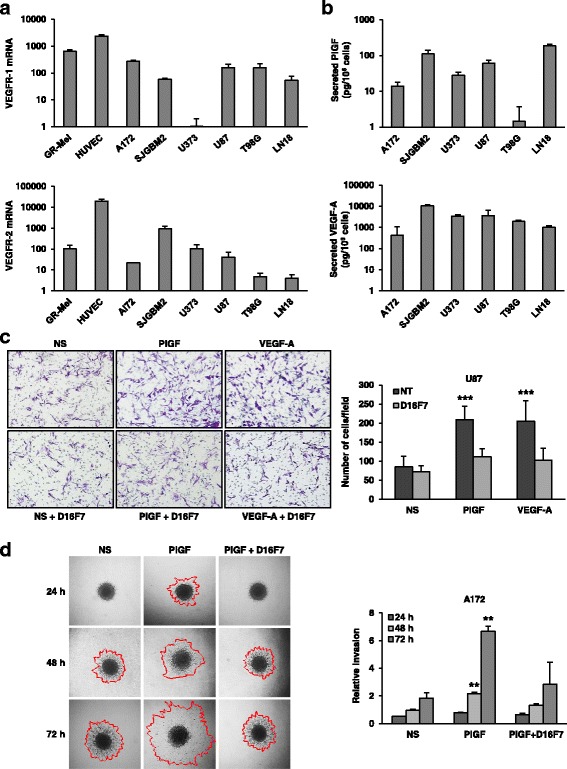
D16F7 inhibitory effects on VEGF-A or PlGF-induced migration and ECM invasion in human GBM cells expressing VEGFR-1. **a** Detection of VEGFR-1 and VEGFR-2 transcripts in GBM cell lines was performed by qRT-PCR. Results indicate relative mRNA expression and are the mean ± SD of three independent determinations. **b** PlGF and VEGF-A secretion was quantified by ELISA (mean ± SD, *n* = 3). **c** Migration of U87 cells in response to PlGF or VEGF-A was evaluated in the absence (not treated, NT) or presence of 5 μg/ml D16F7; NS, non-stimulated cells. Representative photographs of U87 cells are shown (100× magnification). Histograms represent the mean ± SD (*n* = 3) of migrated cells/microscopic field. Results of statistical analysis using one-way ANOVA, followed by Bonferroni’s post-test were as follows: PlGF *vs* NS, PlGF *vs* D16F7 or PlGF *vs* PlGF + D16F7 and VEGF-A *vs* NS, VEGF-A *vs* D16F7 or VEGF-A *vs* VEGF-A + D16F7, *p* < 0.001 (***); differences between NS, D16F7, PlGF + D16F7 or VEGF-A + D16F7 were not significant. **d** For spheroid invasion assay A172 cells were embedded in matrigel in the absence or presence of D16F7 (10 μg/ml) and PlGF (50 ng/ml). Representative pictures of spheroids taken at 24, 48 and 72 h after embedding cells in matrigel (40× magnification) are shown. NS, non-stimulated cells. Relative invasion was quantified as spheroid area difference (in mm^2^) at each of the indicated time points minus day 0. Data are expressed as mean ± SD of triplicate samples and results of statistical analysis were as follows: PlGF *vs* NS and PlGF *vs *PlGF + D16F7 at 48 and 72 h, *p* < 0.01 (**). Differences between NS and PlGF + D16F7 were not significant

With the purpose of analyzing the effect of the anti-VEGFR-1 D16F7 mAb in GBM models, a set of human GBM cell lines was characterized for the expression of membrane VEGFRs and production of VEGFR-1 ligands. The VEGFR-1 mRNA, evaluated by qRT-PCR, was detected in five out of six cell lines (Fig. 1a, upper panel), even though at lower levels compared with the positive control HUVEC. In the same analysis, VEGFR-2 mRNA was observed in all cell lines, although in most of them at a lower level with respect to VEGFR-1 (Fig. 1a, lower panel). ELISA analysis of PlGF and VEGF-A secretion in culture supernatants collected from the different GBM cell lines revealed that: a) PlGF was produced by most of the cell lines tested and was nearly undetectable in T98G cells (Fig. 1b, upper panel), and b) all cell lines secreted substantial amounts of VEGF-A (Fig. 1b, lower panel).

### The anti-VEGFR-1 D16F7 mAb inhibits GBM cell migration and extracellular matrix (ECM) invasion in response to VEGF-A and PlGF

Activation of VEGFR-1 is involved in endothelial and monocytic cell migration, and PlGF has a role in controlling cell motility and invasiveness of cultured cancer cells [3]. In this regard, we recently demonstrated that the anti-VEGFR-1 D16F7 mAb inhibits human melanoma chemotaxis in response to PlGF [15]. The influence of D16F7 on GBM migratory response to VEGF-A and PlGF was tested using VEGFR-1-positive U87 and LN18 cells in Boyden chambers containing gelatin coated filters. VEGF-A and PlGF stimulated substantial chemotaxis of U87 cells, and pre-incubation with D16F7 markedly reduced ligand-induced migration (Fig. 1c). On the other hand, a murine IgG1 control did not affect migration of U87 cells upon stimulation of VEGFR-1 (Additional file 3: Figure S3), in accordance with a previous study with melanoma cells [15]. These data indicated a prevalent role for VEGFR-1 in the promotion of GBM cell migration compared to VEGFR-2, even when VEGF-A was used as stimulus. The anti-VEGFR-1 mAb also inhibited the migratory response of LN18 cells (data not shown). The influence of D16F7 on VEGF-A and PlGF-induced chemotaxis of other VEGFR-1 positive cell lines (i.e., A172 and T98G) could not be tested in this assay because cells failed to adhere to gelatin-coated filters. Therefore, the ability of D16F7 to reduce ECM invasion of A172 cells was tested by a spheroid invasion assay. The VEGFR-1-selective ligand PlGF stimulated matrigel-embedded spheroids to markedly invade the surrounding matrix in a time-dependent manner, and D16F7 significantly reduced GBM cell invasiveness (Fig. 1d). Notably, D16F7 at the concentrations tested in the chemotaxis and invasion assays did not inhibit proliferation in vitro in any of the GBM cell lines tested (data not shown).

### The anti-VEGFR-1 D16F7 mAb inhibits migration of VEGFR-1 positive human GSCs in response to VEGF-A and PlGF

**Fig. 2:**
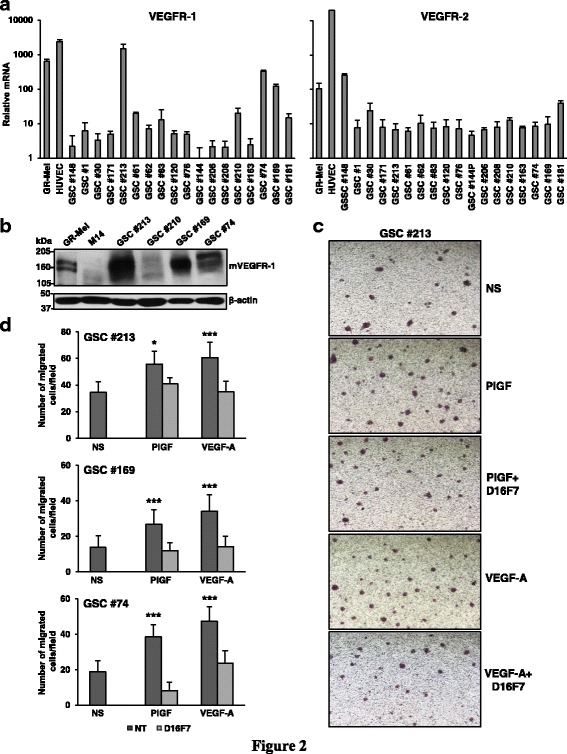
D16F7 inhibitory effects on VEGF-A or PlGF-induced migration of GSCs. **a** Detection of VEGFR-1 and VEGFR-2 transcripts in GSC lines was performed by qRT-PCR. Results indicate relative mRNA expression and are the mean ± SD of three independent determinations. **b** VEGFR-1 protein levels were analyzed by Western blotting using β-actin detection as loading control. HUVEC and M14 cells were used as positive and negative controls, respectively. **c** Migration of GSCs in response to PlGF or VEGF-A in the absence or in the presence of 5 μg/ml D16F7 was analyzed as described in Fig. 1c legend. Photographs from a representative experiment out of three with #213 cells are shown (40× magnification). **d** Histograms represent the mean ± SD (*n* = 3) of migrated cells/microscopic field. Results of statistical analysis using one-way ANOVA, followed by Bonferroni’s post-test were as follows: in #213, PlGF *vs* NS or PlGF *vs* PlGF + D16F7, *p* < 0.05 (*); VEGF-A *vs* NS or VEGF-A *vs* VEGF-A + D16F7, *p* < 0.001 (***); for #169, PlGF *vs *NS or PlGF *vs *PlGF + D16F7 and VEGF-A *vs* NS or VEGF-A *vs* VEGF-A + D16F7, *p* < 0.001 (***); in #74, PlGF *vs* NS or PlGF *vs* PlGF + D16F7 and VEGF-A *vs* NS or VEGF-A *vs* VEGF-A + D16F7, *p* < 0.001 (***). Differences between NS and PlGF + D16F7 or VEGF-A + D16F7 were not statistically significant

GBMs harbor a subset of GBM stem-like cells (GSCs) which are radioresistant and chemoresistant, participate in tumor neovascularization, and promote tumor recurrence [30, 41]. To assess whether VEGFR-1 plays a role in the aggressive behavior of this GBM cell subset, 18 patient-derived GSC lines (Table 2) were analyzed for VEGFR-1 and VEGFR-2 expression by qRT-PCR. VEGFR-1 expression was quite heterogeneous, while all GSC lines were weakly positive for VEGFR-2 (Fig. 2a). VEGFR-1 expression was verified by Western blotting (Fig. 2b) in GSCs expressing the greatest levels of VEGFR-1 transcript. VEGFR-1 positive GSC lines #213, #169 and #74 migrated in response to both VEGF-A and PlGF, and treatment with D16F7 reverted ligand-induced chemotaxis (Fig. 2c and d).

**Table 2:** Characteristics of the original GBMs from which GSCs were derived

GSC line	Tumor location	Primary (P) Recurrent (R)	Overall survival (months)	EGFRvIII
#1	Temporal	P	12,5	Neg
#30	Frontal	P	7,5	Pos
#61	Occipital	P	6,0	Pos
#62	Frontal	R	14,0	Neg
#74	Frontal	P	8,0	Pos
#76	Frontal	P	42,0	Neg
#83	Temporal	P	8,0	Pos
#120	Parietal	R	16,5	Neg
#144	Temporal	P	26,0	Pos
#148	Parietal	R	8,0	Neg
#163	Parietal	P	2,0	Neg
#169	Temporal	P	9,0	Neg
#171	Frontal	R	17,0	Pos
#181	Occipital	R	17,0	Pos
#206	Temporal	P	27,0	Neg
#208	Temporal	R	33,0	Neg
#210	Parietal	P	10,5	Pos
#213	Frontal	R	10,5	Neg

**Fig. 3:**
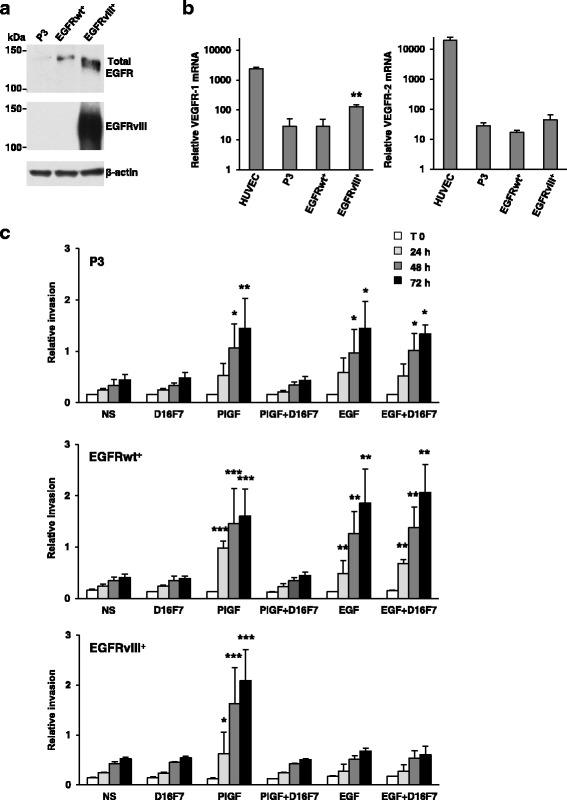
D16F7 inhibits ECM invasion of P3, EGFRwt^+^ and EGFRvIII^+^ GSCs that express VEGFR-1. **a** EGFR or mutated EGFRvIII protein levels were analyzed by Western blotting using β-actin detection as loading control. **b** Detection of VEGFR-1 and VEGFR-2 transcripts was performed by qRT-PCR. Results indicate relative mRNA expression and are the mean ± SD of three independent determinations. EGFRvIII^+^ *vs* P3 and EGFRwt^+^ cells, *p* < 0.01 (**). **c** For spheroid invasion assay cells were embedded in matrigel in the absence or presence of D16F7 (10 μg/ml) and PlGF or EGF. Data are the mean ± SD (*n* = 5–10) relative invasion evaluated as described in Fig. 1d legend. Results of statistical analysis using one-way ANOVA, followed by Bonferroni’s post-test were as follows: in P3 cells PlGF *vs* NS, PlGF *vs* D16F7 or PlGF *vs* PlGF + D16F7, *p* < 0.05 (*) at 48 h and *p* < 0.01 (**) at 72 h; in EGFRwt^+^ cells PlGF *vs* NS, PlGF *vs* D16F7 or PlGF *vs* PlGF + D16F7, *p* < 0.001 (***) at 24, 48 and 72 h; in EGFRvIII^+^ cells PlGF *vs* NS, PlGF *vs* D16F7 or PlGF *vs* PlGF + D16F7, *p* < 0.05 (*) at 24 h and *p* < 0.001 (***) at 48 and 72 h. In all cell lines differences between NS and D16F7 or PlGF + D16F7 or between EGF and EGF + D16F7 were not significant. In P3 cells, EGF *vs *NS or EGF + D16F7 *vs* NS, *p* < 0.05 (*) at 48 and 72 h; in EGFRwt^+^ cells, EGF *vs* NS or EGF + D16F7 *vs* NS, *p* < 0.01 (**) at all time points

Mutation/amplification of EGFR has been reported in a wide proportion of GBMs. In fact, a large-scale sequencing study indicated that 57% of GBM patient samples contain mutation, rearrangement, altered splicing, and/or focal amplification of EGFR and that various mutations often co-occur with EGFR rearrangement and/or amplification [42]. In this context, we next investigated the influence of D16F7 mAb in a patient-derived GSC line (P3) modified to over-express wild-type EGFR (EGFRwt^+^) or mutant EGFR (ligand binding domain-deficient EGFRvIII^+^) (Fig. 3a). These GSCs are characterized by invasive or angiogenic in vivo behavior depending on whether EGFRwt (more invasive) or EGFRvIII (more angiogenic) is overexpressed [31]. Analysis of P3 cells and the two P3-derived cell lines by qRT-PCR revealed that all of them expressed VEGFR-1 as well as VEGFR-2 (Fig. 3b). VEGFR-1 level was significantly higher in EGFRvIII^+^ than in P3 and EGFRwt^+^ cells.

Strikingly, PlGF stimulated ECM invasion in all cell lines in a time-dependent manner while, as expected, only P3 and more significantly EGFRwt^+^ cells responded to EGF (Fig. 3c, Additional file 4: Figure S4, Additional file 5: Figure S5 and Additional file 6: Figure S6). Consistently, treatment with D16F7 mAb inhibited invasion induced by PlGF, but it did not affect ECM invasion in response to EGF (Fig. 3c, Additional file 4: Figure S4, Additional file 5: Figure S5 and Additional file 6: Figure S6).

These results suggest that D16F7 may inhibit the aggressive behavior of GSCs expressing mutated EGFR.

### D16F7 mAb inhibits VEGFR-1 auto-phosphorylation, intracellular signal transduction and ECM invasion in VEGFR-1 transfected GBM cells

**Fig. 4:**
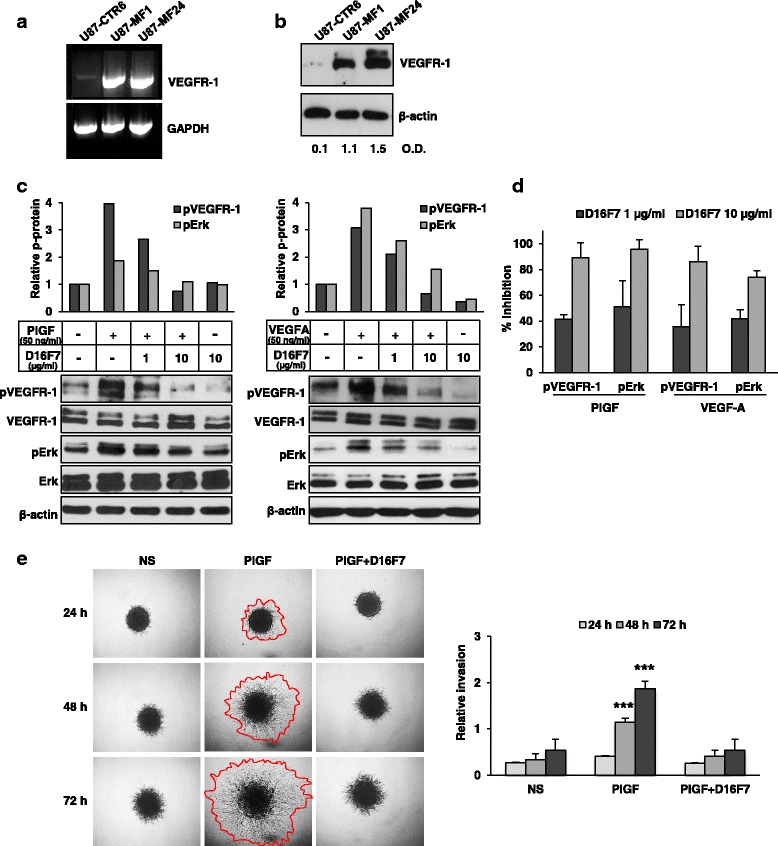
Inhibition by D16F7 of VEGF-A and PlGF-induced phosphorylation of VEGFR-1 at Tyr 1213 in GBM cells over-expressing VEGFR-1. **a** VEGFR-1 mRNA levels in U87-derived clones transfected with control (U87-CTR6) or VEGFR-1 expressing (U87-MF1 and U87-MF24) vectors was analyzed by RT-PCR. Amplified products were separated on 1% agarose gels and results are representative of one out of two different experiments giving comparable results. **b** VEGFR-1 protein levels in U87-derived clones transfected with control or VEGFR-1 expressing vectors were analyzed by Western blotting. Numbers below immunoblot lanes indicate VEGFR-1/β-actin optical density (O.D.) ratios. **c** Western blotting of total or phosphorylated VEGFR-1 (pVEGFR-1) at tyrosine 1213 and total or phosphorylated Erk1/2 (pErk) in untreated or D16F7 (1 or 10 μg/ml) pre-treated U87-MF24 cells in response to PlGF or VEGF-A. Histograms represent the densitometric quantification of band intensities in the corresponding immunoblots, expressed as pVEGFR-1/VEGFR-1 ratio relative to untreated control, after normalization for β-actin expression. Normalized pVEGFR-1/VEGFR-1 or pErk/Erk protein ratio in untreated cells was considered equal to 1. **d** Histogram represents the mean ± SD percentage inhibition values of PlGF or VEGF-A-induced VEGFR-1 phosphorylation or Erk1/2 phosphorylation in U87-MF24 cells after treatment with 1 and 10 μg/ml D16F7, calculated from immunoblot densitometric analysis of three independent experiments. **e** For spheroid invasion assay U87-MF24 cells were embedded in matrigel in the absence or presence of D16F7 (10 μg/ml) and PlGF (50 ng/ml). Representative pictures of spheroids taken at 24, 48 and 72 h after embedding cells in matrigel (40× magnification) are shown; NS, non-stimulated cells. Relative invasion was quantified as described in Fig. 1d legend. Data are expressed as mean ± SD (*n* = 6–10) and results of statistical analysis were as follows: PlGF *vs* NS, PlGF *vs* D16F7 or PlGF *vs* PlGF + D16F7, *p* < 0.001 (***) at 48 and 72 h. Differences between NS and PlGF + D16F7 were not significant

Despite the presence of a conserved kinase domain containing an ATP binding site in VEGFR-1, ligand stimulation of the receptor results in only minor tyrosine phosphorylation in vitro and in vivo [43]. Indeed, the weak kinase activity of the receptor has posed challenges to studying features of VEGFR-1 signal transduction. To investigate the ability of D16F7 to affect signal transduction in GBM cells, we over-expressed the human VEGFR-1 membrane form in U87 expressing little endogenous VEGFR-1 protein. Cells transfected with control or VEGFR-1 cDNA-containing vectors were subsequently analyzed by RT-PCR (Fig. 4a) and Western blotting (Fig. 4b). For further experiments to assess the influence of D16F7 on signal transduction, the clone exhibiting the greatest VEGFR-1 protein expression was tested (i.e., U87-MF24). The Tyr 1213 residue in VEGFR-1 is regarded as one of the major auto-phosphorylation sites responsible for activation of intracellular signaling pathways [44, 45]. Accordingly, we investigated whether receptor stimulation by its ligands (VEGF-A and PlGF) resulted in Tyr 1213 phosphorylation in transfected cells and whether D16F7 could inhibit this effect. Exposure of U87-MF24 (Fig. 4c) cells to VEGF-A or PlGF for 10 min induced robust VEGFR-1 kinase activity, as indicated by a marked increase of protein phosphorylation at Tyr 1213, and D16F7 reduced receptor auto-phosphorylation in a dose-dependent manner. VEGFR-1 activation by VEGF-A or PlGF in normal cells (e.g., endothelial cells, fibroblasts, monocytes) stimulates Erk1/2 of the MAPK signaling pathway [3, 16], which, in turn, promotes tumor cell invasion and migration [46]. Accordingly, we investigated whether VEGF-A- or PlGF-induced receptor auto-phosphorylation at Tyr 1213 in U87-MF24 cells was accompanied by Erk1/2 phosphorylation, and whether D16F7 could reduce Erk1/2 activation. Results revealed that D16F7 markedly inhibited Erk1/2 phosphorylation stimulated by both ligands (Fig. 4c). Quantitative results are summarized as percentage inhibition of VEGFR-1 or Erk1/2 phosphorylation achieved after treatment with D16F7, calculated across three independent experiments (Fig. 4d).

VEGFR-1 over-expression in U87-MF24 cells highly stimulated ECM invasion triggered by PlGF and inhibition of PlGF-induced signaling by D16F7 resulted in abrogation of ECM invasion (Fig. 4e).

## Discussion

In the present study we demonstrate for the first time that the novel anti-VEGFR-1 mAb D16F7, which diminishes receptor activation by VEGF-A and PlGF, inhibits chemotaxis and ECM invasion of human GBM and patient-derived GSC lines.

Our data suggest that VEGFR-1 itself can transmit signals that promote GBM cell invasiveness. Importantly, since D16F7 does not reduce VEGFR-1 interaction with its ligands while inhibiting receptor homodimerization, the mAb is considered to display inhibitory effects on VEGFR-1 activation in a non-competitive fashion [15]. Moreover, D16F7 does not hamper soluble VEGFR-1 ability to act as decoy receptor for VEGF-A and PlGF. This is particularly important considering the role of the soluble receptor in controlling tumor progression. In fact, in GBM low soluble VEGFR-1/VEGF-A ratio has been related to higher aggressiveness compared with astrocytomas [47].

Characterization of GBM lines showed that VEGF-A and PlGF are secreted by most of the cell lines tested, suggesting that an autocrine loop may occur in VEGFR-1 expressing GBMs through activation of the receptor tyrosine kinase activity, in accordance with a previous study [39]. Indeed, since we found that VEGFR-1 is frequently detected in GBM specimens, D16F7 is expected to interrupt the autocrine loop that favors tumor aggressiveness.

Although required for inflammatory reactions associated with tumor growth and metastasis and for monocyte migration [48, 49], VEGFR-1 kinase activity is weakly induced upon ligand binding and receptor signaling has not been fully elucidated in tumor cells [43]. Potential tyrosine phosphorylation sites have been identified in VEGFR-1 [17, 44] and their role in receptor activation in GBM has been only recently investigated [50]. Tyrosine 1213, which is regarded as the main auto-phosphorylation site responsible for activation of intracellular pathways [9, 44, 45], became phosphorylated in a highly VEGFR-1-expressing GBM cell line upon exposure to exogenous VEGF-A or PlGF [50]. In our study with U87-derived cells over-expressing VEGFR-1, exposure to VEGF-A or PlGF causes substantial receptor phosphorylation at tyrosine 1213 and pre-treatment with D16F7 prevents VEGFR-1 auto-phosphorylation in response to both ligands. Conversely, it has been reported that an anti-PlGF antibody only partially affected growth factor-induced VEGFR-1 auto-phosphorylation at this amino acid residue [50]. Therefore, our data strongly suggest that blockage of VEGFR-1 activity is more efficiently achieved using D16F7 mAb, which avoids receptor activation by both VEGF-A and PlGF. Moreover, in our model VEGFR-1 auto-phosphorylation is followed by downstream phosphorylation of Erk1/2 that is counteracted by D16F7 treatment.

Analysis of VEGFR-1 in GSC lines indicates a quite variable expression of the receptor. In particular, ~17% (3 out of 18) of the GSCs tested demonstrate high levels of VEGFR-1 transcript that result in remarkable amounts of the corresponding protein detected on immunoblot. In this context, our results are in line with a recent study in a limited number of patient-derived GSC samples showing VEGFR-1 staining by immunocytochemistry analysis [40].

The specificity of D16F7 against the chemotactic and invasive response to VEGF-A and PlGF was confirmed by the lack of antibody activity against cells responding to ligands that do not bind VEGFR-1 (i.e. EGF). Meanwhile, D16F7 markedly inhibits VEGFR-1 ligand-induced motility of GSCs expressing mutant EGFR (i.e., #74 and P3-derived EGFRvIII^+^ GCSs) as well as GSCs over-expressing EGFRwt (i.e., P3-derived EGFRwt^+^ GSCs). Actually, GBM cells harboring EGFRvIII mutation have recently been found to possess an angiogenic phenotype in vivo due to upregulated secretion of VEGF-A compared with cells over-expressing EGFRwt, which instead showed an enhanced invasive behavior [31].

Tumor cell invasion, angiogenesis, and genetic intra-tumor heterogeneity are hallmarks of GBM that reflect major factors involved in treatment failure [51–54]. Indeed, EGFR amplification and mutation are invariably expressed in a heterogeneous manner, and the presence of EGFRvIII in a minor population of GBM cells has been shown to confer a more aggressive tumor phenotype through paracrine mechanisms [53]. It has recently been demonstrated that EGFR amplification is an early event in GBM development, while EGFRvIII subsequently emerges during disease progression to drive a more aggressive tumor that becomes dependent on angiogenesis for growth [31]. Moreover, studies performed in a large cohort of GBM patients have indeed shown that VEGFR-1 is detected in tumor vessels and at significantly higher levels compared with lower grade gliomas [55]. In this context, since D16F7 can interact with VEGFR-1 expressed by tumor cells as well as by endothelial cells, the advantage of D16F7 in the control of GBM growth is two-fold: the mAb may inhibit tumor cell invasion and angiogenesis.

VEGF-A and PlGF produced by GBM cells can also stimulate angiogenesis and induce accumulation of VEGFR-1-positive bone marrow-derived myeloid cells in glioma tissues [24]. These cells are involved in neovessel formation and ECM invasion by secreting MMP and angiogenic factors or other cytokines that promote tumor cell survival [24, 56]. Since D16F7 is able to suppress bone marrow mobilization of myeloid progenitors [15], this property may additionally contribute to restraining GBM progression.

## Conclusions

The results presented indicate that VEGFR-1 is an appropriate target for reducing GBM aggressiveness and that D16F7-derived anti-VEGFR-1 humanized mAbs warrant further investigation for therapeutic intervention of GBM. Due to VEGFR-1 limited involvement in physiological angiogenesis, D16F7-derived molecules may synergize with agents targeting VEGFR-2/VEGF-A or EGFR/EGF without additional systemic toxicity.

## Additional files

Additional file 1: Figure S1. Immunohistochemical analysis of VEGFR-1 expression in GBM tissue sections. Representative images are presented (25× magnification). VEGFR-1 immunostaining was scored as described in Table 1: score 1 (<10%); score 2 (11–25%); score 3 (26–50%); score 4 (51–75%); 5 (>75%). (PDF 484 kb)

Additional file 2: Figure S2. VEGFR-1 immunostaining of endothelial and tumor cells in GBM tissue. Representative image from a GBM tissue section showing VEGFR-1 staining in endothelial cells (red arrows), along with tumor cells (black arrows). (PDF 60 kb)

Additional file 3: Figure S3. Specificity of D16F7 inhibitory activity on GBM cell migration in response to VEGFR-1 activation. Migration of U87 cells in response to PlGF (50 ng/ml) was evaluated in the presence of D16F7 or of a murine IgG1 control mAb (5 μg/ml). Histogram represents the mean (± SD) percentage inhibition of cell migration calculated from 3 independent determinations. (PDF 20 kb)

Additional file 4: Figure S4. Inhibition of ECM invasion by D16F7 cells in a spheroid assay with P3 cells. Representative pictures of spheroids taken at 24, 48 and 72 h after embedding P3 cells in matrigel (40× magnification) and referring to the experiment described in Fig. 3c legend. (PDF 255 kb)

Additional file 5: Figure S5. Inhibition of ECM invasion by D16F7 cells in a spheroid assay with EGFRwt^​+^ cells. Representative pictures of spheroids taken at 24, 48 and 72 h after embedding EGFRwt^+^ cells in matrigel (40× magnification) and referring to the experiment described in Fig. 3c legend. (PDF 268 kb)

Additional file 6: Figure S6. Inhibition of ECM invasion by D16F7 cells in a spheroid assay with EGFRvIII^​+^ cells. Representative pictures of spheroids taken at 24, 48 and 72 h after embedding EGFRvIII^+^ cells in matrigel (40× magnification) and referring to the experiment described in Fig. 3c legend. (PDF 198 kb)

## Abbreviations

EGF: Epidermal growth factor
EGFR: Epidermal growth factor receptor
FBS: Fetal bovine serum
GBM: Glioblastoma
GSCs: Glioblastoma stem cells
HUVEC: Human umbilical vascular endothelial cells
mAbs: Monoclonal antibodies
MAPKs: Mitogen-activated protein kinases
MMP: Matrix metalloproteinase
MTS: 3-(4,5-dimethylthiazol-2-yl)-5-(3-carboxymethoxyphenyl) 2-(4-sulphophenyl)-2H–tetrazolium, inner salt
NBM: Neurobasal medium
PBS: Phosphate-buffered saline
PlGF: Placental growth factor
qRT-PCR: Quantitative real-time reverse transcriptase –polymerase chain reaction
VEGFR-1: Vascular endothelial growth factor receptor-1
