# Supplementary figures and images for: The anti-vascular endothelial growth factor receptor-1 monoclonal antibody D16F7 inhibits invasiveness of human glioblastoma and glioblastoma stem cells
Source: J Exp Clin Cancer Res. 2017 Aug 10;36:106. doi: 10.1186/s13046-017-0577-2 (PMC5553938; doi:10.1186/s13046-017-0577-2)

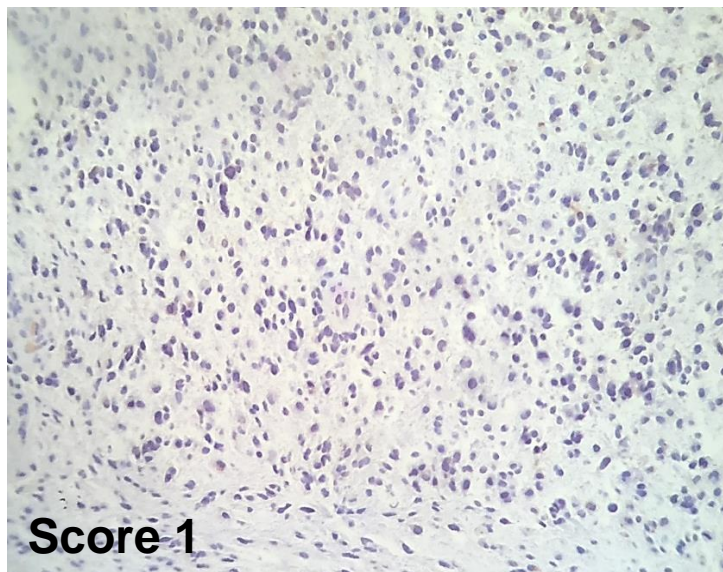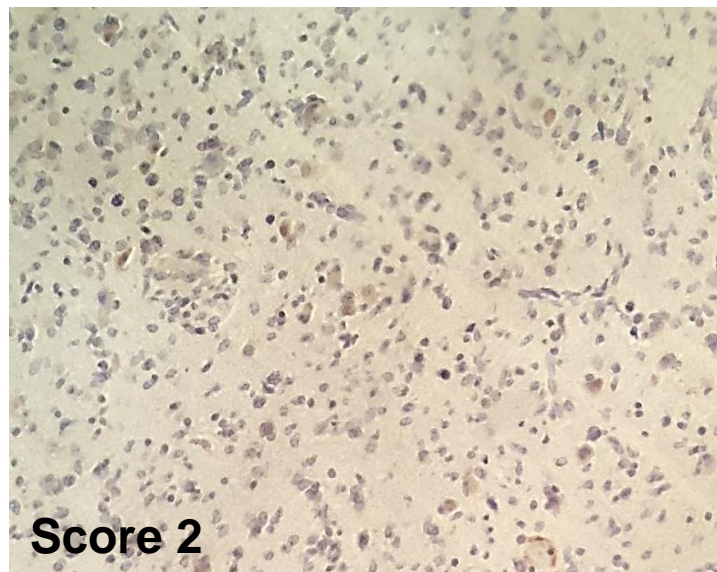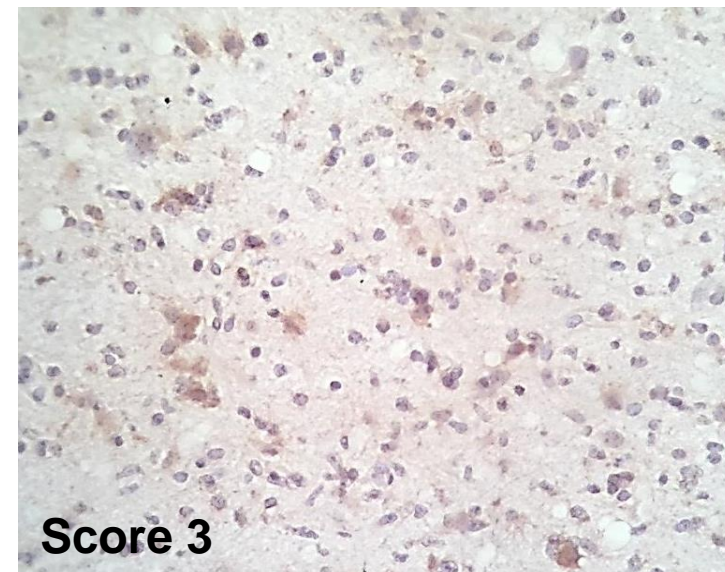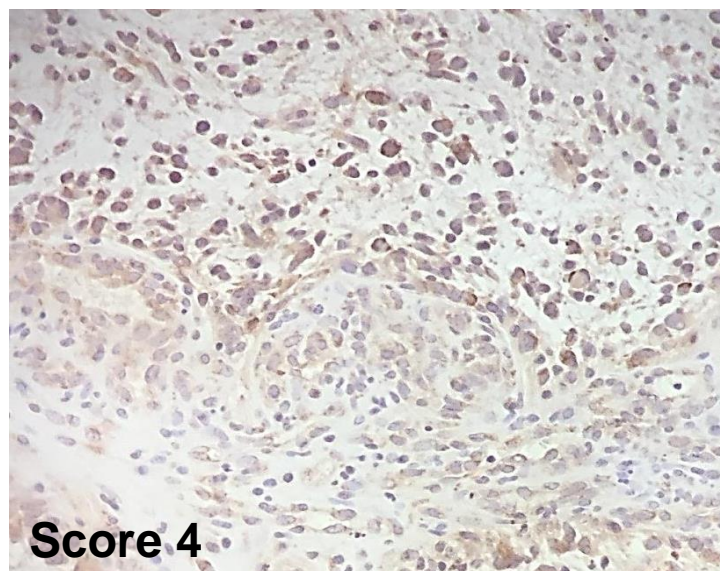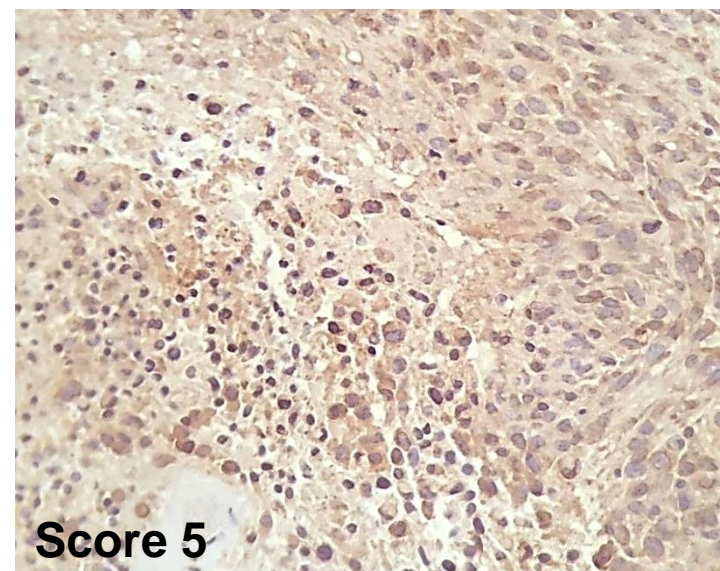

**Figure S1**

Supplement: Supplementary file 1 — Immunohistochemical analysis of VEGFR-1 expression in GBM tissue sections. Representative images are presented (25× magnification). VEGFR-1 immunostaining was scored as described in Table 1: score 1 (<10%); score 2 (11–25%); score 3 (26–50%); score 4 (51–75%); 5 (>75%). (PDF 484 kb) [file 13046_2017_577_MOESM1_ESM.pdf]

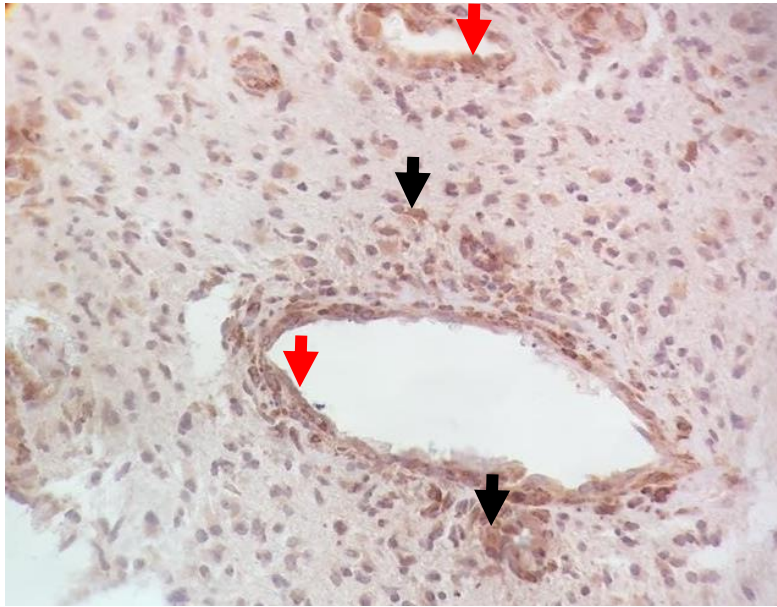

**Figure S2**

Supplement: Supplementary file 2 — VEGFR-1 immunostaining of endothelial and tumor cells in GBM tissue. Representative image from a GBM tissue section showing VEGFR-1 staining in endothelial cells (red arrows), along with tumor cells (black arrows). (PDF 60 kb) [file 13046_2017_577_MOESM2_ESM.pdf]

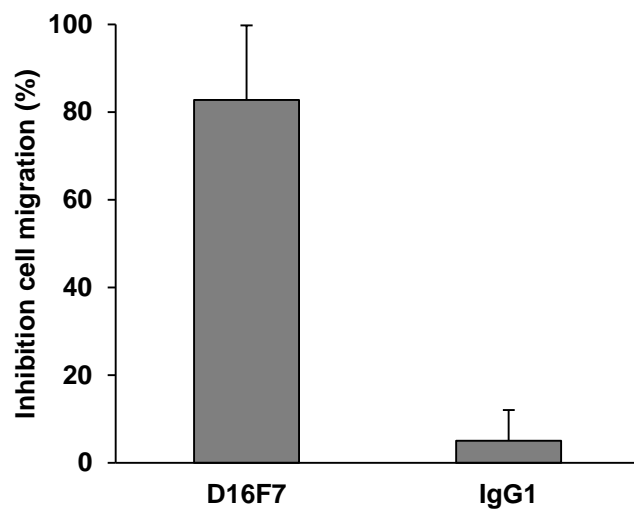

**Figure S3**

Supplement: Supplementary file 3 — Specificity of D16F7 inhibitory activity on GBM cell migration in response to VEGFR-1 activation. Migration of U87 cells in response to PlGF (50 ng/ml) was evaluated in the presence of D16F7 or of a murine IgG1 control mAb (5 μg/ml). Histogram represents the mean (± SD) percentage inhibition of cell migration calculated from 3 independent determinations. (PDF 20 kb) [file 13046_2017_577_MOESM3_ESM.pdf]

P3

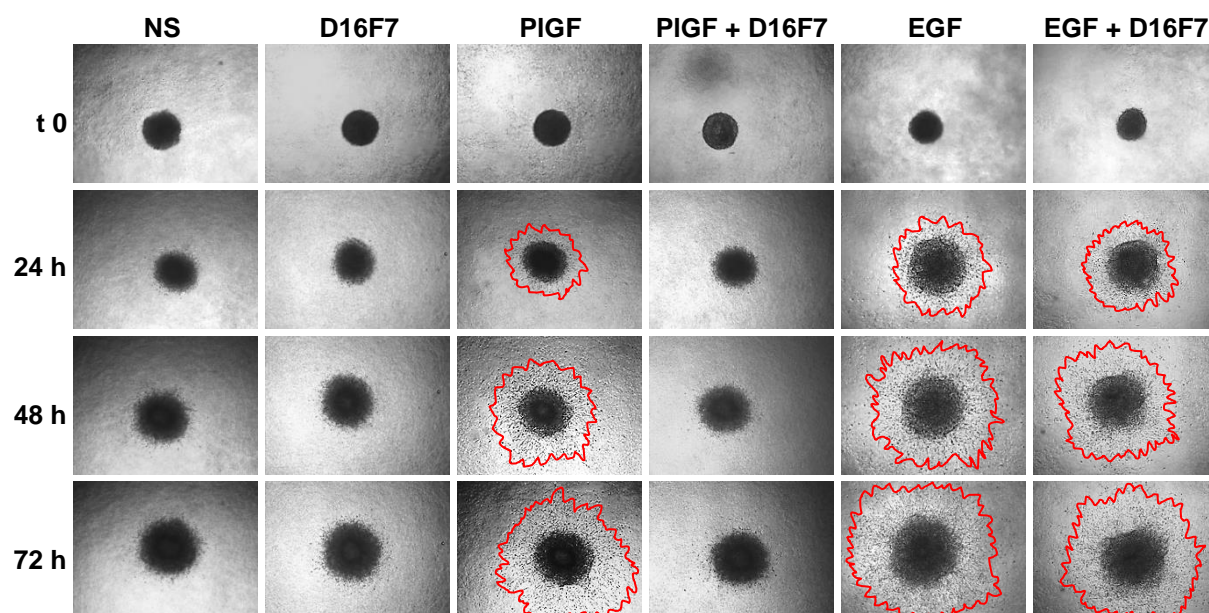

Figure S4

Supplement: Supplementary file 4 — Inhibition of ECM invasion by D16F7 cells in a spheroid assay with P3 cells. Representative pictures of spheroids taken at 24, 48 and 72 h after embedding P3 cells in matrigel (40× magnification) and referring to the experiment described in Fig. 3c legend. (PDF 255 kb) [file 13046_2017_577_MOESM4_ESM.pdf]

**EGFRwt<sup>+</sup>**

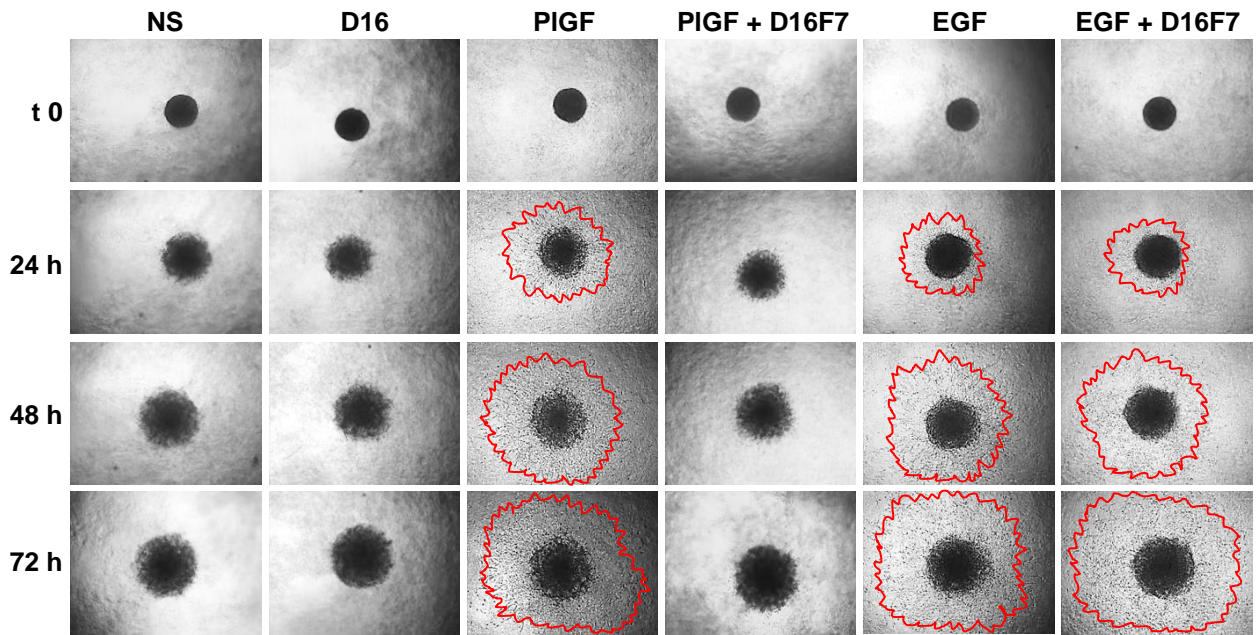

**Figure S5**

Supplement: Supplementary file 5 — Inhibition of ECM invasion by D16F7 cells in a spheroid assay with EGFRwt​+ cells. Representative pictures of spheroids taken at 24, 48 and 72 h after embedding EGFRwt+ cells in matrigel (40× magnification) and referring to the experiment described in Fig. 3c legend. (PDF 268 kb) [file 13046_2017_577_MOESM5_ESM.pdf]

EGFRvIII<sup>+</sup>

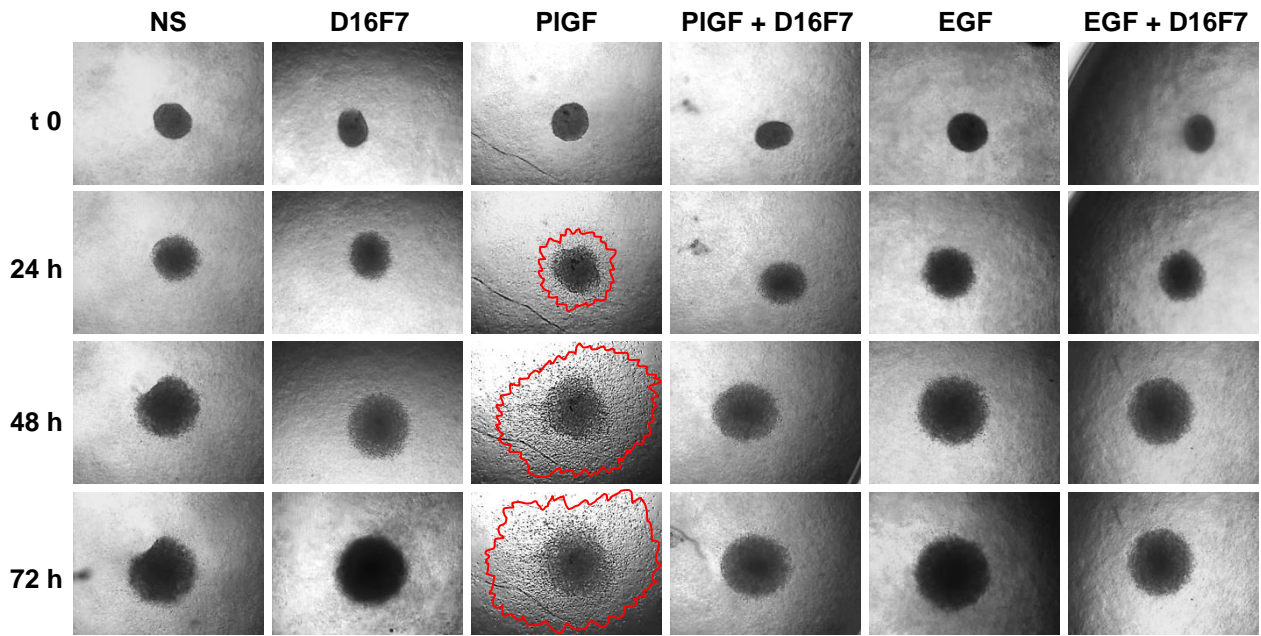

Figure S6

Supplement: Supplementary file 6 — Inhibition of ECM invasion by D16F7 cells in a spheroid assay with EGFRvIII​+ cells. Representative pictures of spheroids taken at 24, 48 and 72 h after embedding EGFRvIII+ cells in matrigel (40× magnification) and referring to the experiment described in Fig. 3c legend. (PDF 198 kb) [file 13046_2017_577_MOESM6_ESM.pdf]
